# The Mystery of Infection

**Published:** 1915-12

**Authors:** G. Henri Bogart

**Affiliations:** Shelbyville, Ind.


### The Mystery of Infection.

BY DR. G. HENRI BOGART, SHELBYVILLE, ILL.

So often the case of contagious disease, particularly pneumonia, scarlatina or diphtheria springs up with no known source of infection apparent that sometimes we are inclined to believe in the sporadic inception of disease, in spontaneous generation of germs, or even in the absence of necessity of germ production of the disease in question.

Even though the physician is not assailed by any of these doubts, he has the opinion of the friends and family to encounter, well meaning folks who are positive that the patient had been nowhere to be exposed, and that there have been no cases in the community.

The doctor's pleas or commands for care and quarantine are likely to meet with half-hearted acceptance, if not with total disregard.

The more intelligent of his clients will be most likely to make use of the little learning that is a dangerous thing.

The doctor's power for good, his influence and his personal business success all depend largely on the confidence of the community.

Whatever weakens that confidence weakens him. There will be there the many who having "faith in things not seen" will follow him to the end of the way, but he wants, he needs, and he ought to have the unqualified support of the belief of his clientele; and he wants the supreme confidence in himself and in his conclusions.

To determine this matter, Surgeon General Blue of the U. S. Public Health Service conducted a series of investigations during an epidemic of diphtheria in Detroit. Cloaked with authority from the City Board of Health, his field men went, street by street, in various quarters of the city. Every resident had smears taken from throat and nose, to the number of 4093 persons apparently in good health. A competent force of bacteriologists worked regularly and steadily on the 8758 smears taken and found 39 diphtheria carriers, immune to any macroscopic appearance or symptoms of the disease.

That means that practically 1 per cent of the population were unconscious and immune diphtheria carriers, or a total of 5376 in the city.

Every one of these carriers was capable of infecting the susceptible whom they met on the street, in the public conveyance or in the place of amusement. It were a very humble movie, theater, school or congregation that did not include at least one source of infection, one active center of contagion.

The mystery of the past becomes as simple as breathing under this severely scientific investigation.

Armed with this official solution, the physician should be able to explain the facts concerning the absolutely universal source of all communicable diseases.

On the other hand, he wants to avoid the germophobiac who is inclined to keep his doctor on the guess and the jump anyhow. One of these fellows who had tormented his medical attendant into various frenzies managed to die anyhow, and provided in his will that his body be hauled to the grave in a nice, clean, open wagon, because a hearse was certain to be infected with germs.

Relatives sought to have the will set aside on the grounds of mental unsoundness, as shown by the last testament, but the Supreme Court of Indiana sustained it.

There is one point in practice in which the profession should take more interest; i. e., the relative idiosyncratic individual powers of withstanding many of these ailments.

In scarlet fever we see one virulent case; others in various degrees of what used to be called "only scarlet rash," while other members of the family go free from visible evidence of attack.

And yet the most immune of them all may be the angel of death for some other household.

---

### Dr. E. Mercado.

Dr. E. Mercado, a Filipino physician, believes he has demonstrated the efficacy of a new cure for leprosy, hitherto believed to be an incurable disease. The treatment is with chaulmoogra oil, made from takatogenos kurzil seeds. The treatment has been used by others, and in the *London Lancet* last year record was made of the discharge by Dr. Victor G. Heiser from the San Labaro Hospital for Lepers at Manila of two patients so treated who were declared cured. Dr. Mercado has discharged from the great leper colony at Culion, P. I., twenty-three patients who he declares are completely cured of leprosy.

